# Trading upon a Name

**Published:** 1887-09

**Authors:** Ad. Lippe

**Affiliations:** Philadelphia


### TRADING UPON A NAME.

AD. LIPPE, M. D., PHILADELPHIA.

The American Institute of Homœopathy, at its session in 1886, indorsed fully the article on "Homœopathy," by H. H. Furness, Esq., as published in the *Encyclopædia Americana.* In that article a lucid exposition of the true philosophy of Homœopathy was given, and the intelligent portion of the profession and of the people took it for granted that all homœopathists practiced in accordance with the rules laid down by Samuel Hahnemann for applying the natural and universal law of the similars for the cure of the sick.

The President of the Institute, in his annual address, June 27th, 1887, has apparently utterly ignored the teachings of Hahnemann indorsed but a year ago, and he fully recommends the *trading upon a name.* There is large latitude left to the members of the Institute to avail themselves of the new discoveries made by the "Regulars" since Hahnemann's days; there is a long list of palliatives added much more pernicious than the former use of Opium, and certainly not to be thought of any more than was the once highly praised painkiller, Opium. It is our present object to enlighten these misguided *traders upon a name,* and show them, from a circumstance just come to our knowledge, where these "traders" will be left.

The author of the article on "Homœopathy," for which the Institute so politely thanked him in 1886, received a few days ago a letter from a leading gentleman in New York. He desires to learn the name and residence of a trustworthy homœopathic physician in New York. His experience with so-called medical men who *trade on a name* is a sad one. On a visit to England his son had an attack of typhus fever. By full conviction the parents were homœopathists, but in London they unfortunately fell into the hands of a "trader." More traders were called in. The treatment differed essentially from the treatment of former homœopaths. The traders talked of scientific progress since Hahnemann's day, but the son died. Their faith in Homœ-

opathy was shaken, and when, on their return to the United States, another son was attacked by the same disease, allopathists were consulted, and that son also died. Convinced that the first son was treated unhomœopathically by a "trader," it dawned on them that it was a terrible mistake to trust to a man who *trades on a name.* Being of a logical mind, their inquiry was: Where can we find a trustworthy physician who will practice Homœopathy as the master taught it and as it was practiced by the founders of the Institute?

Be it remembered, Homœopathy was made a success and gained a name because these founders followed the master's directions implicitly.

*Trading on that name* without following the master's injunctions is like the play of Hamlet with Hamlet left out, and no sophistry of even a President of the Institute can make it otherwise. Only one case of many has been here related merely to show to the "*traders on a name*" the handwriting on the wall.

